# Sleep Duration and Mortality in Japan: the Jichi Medical School Cohort Study

**DOI:** 10.2188/jea.14.124

**Published:** 2005-03-18

**Authors:** Yoko Amagai, Shizukiyo Ishikawa, Tadao Gotoh, Yuriko Doi, Kazunori Kayaba, Yosikazu Nakamura, Eiji Kajii

**Affiliations:** 1 Division of Community and Family Medicine, Center for Community Medicine, Jichi Medical School.; 2 Wara National Health Insurance Hospital.; 3 Department of Epidemiology, National Institute of Public Health.; 4 Department of Nursing, School of Health and Social Services, Saitama Prefectual University.; 5 Department of Public Health, Jichi Medical School.

**Keywords:** cohort studies, mortality, sleep, cause of death, Japan

## Abstract

BACKGROUND: Although sleep is one of the most important health-related factors, relationship between sleep duration and mortality has not been fully discussed.

METHODS: Study subjects were 11,325 participants (4,419 males and 6,906 females) in the Jich Medical School Cohort Study, a population-based prospective study. Baseline data were obtained by questionnaire and health checkups between April 1992 and July 1995 in 12 rural areas in Japan. Main outcome measures were all-cause and cause-specific mortality derived from death certificates up to December 31, 2001. Cox’s proportional hazard models were applied to analyze the association of sleep duration with mortality.

RESULTS: A total of 495 deaths (289 males and 206 females) were observed during the average of 8.2-year follow-up period. After adjusting for age, systolic blood pressure, serum total cholesterol, body mass index, smoking habits, alcohol drinking habits, education, and marital status, the hazard ratios (95% confidence intervals) of all-cause mortality for individuals sleeping shorter than 6 hours and 9 hours or longer were 2.4 (1.3-4.2) and 1.1 (0.8-1.6) in males, and 0.7 (0.2-2.3) and 1.5 (1.0-2.4) in females, respectively, relative to those with 7-7.9 hours sleep.

CONCLUSION: Our data suggest that males with short sleep and females with long sleep were at an elevated risk of death.

Sleep is one of the most important factors contributing to health. Although there were some studies examining the relationship between sleep duration and mortality,^1^^-^^10^ it has not been fully discussed. Most previous reports have found that there was a U-shaped relationship between sleep duration and all-cause mortality in both males and females.^1^^-^^4^^,^^6^

The purpose of the present study was to examine whether sleep duration is associated with all-cause mortality and cause-specific mortality in the general Japanese population.

## METHODS

We used the data set of the Jichi Medical School Cohort Study, which is a population-based prospective study investigating risk factors for cardiovascular diseases started in 1992.^11^ The baseline data were obtained between April 1992 and July 1995 in 12 rural areas in Japan. Mass screening examinations for cardiovascular diseases have been conducted since 1983 in accordance with the health and medical service law for the aged, and we used this system to collect the data. In each community, a local government office sent personal invitations to all the subjects by mail. The subjects for the mass screening examinations were residents aged 40-69 years in 8 areas and were 30 years and older in one area. Subjects for other age groups were included in 3 areas. As a result, 12,490 subjects (4,913 males and 7,577 females) were eligible for all ages (19-93 years of age). The overall response rate was 65.4%. Subjects for the present study were 11,325 persons (4,419 males and 6,906 females) with data of sleep duration but without history of myocardial infarction, stroke, and malignant neoplasms at baseline.

Health checkup was carried out in each community. Body height and body weight were measured, then body mass index (BMI) was calculated as weight (kg) / height (m)^2^. Systolic blood pressure (SBP) and diastolic blood pressure (DBP) were measured with a fully automated sphygmomanometer. Serum total cholesterol and high-density lipoprotein cholesterol were also measured with standard methods.

Information about medical history and sociodemographic characteristics was obtained by trained interviewers using a standardized questionnaire. Smoking status was judged as current smoker, ex-smoker, or never smoked, and alcohol drinking status was judged as current drinker, ex-drinker, or never drank. For educational level, age at finished education was asked. Age at finished education was categorized as age<15 years, 15-17, and 18+ years. Marital status was judged as having a spouse or not. Interviewers directly asked each participant about ordinary daily schedules. Participants were asked what time they usually fell asleep at night and awoke in the morning. This enabled us to calculate sleep duration.

Written informed consent for the study was obtained individually for the responders of the mass screening health check-up, and all the responders agreed to join the study.

Causes of death were identified with death certificates, which were collected at the respective local public health centers with permission from the Ministry of General Affairs and the Ministry of Health, Labor and Welfare. The people who moved out of the communities during the observation period were followed up until the date of the emigration. Data on emigration of the study subjects were obtained every year from their municipal governments.

The difference in mean sleep duration between males and females was tested by an unpaired t-test. Associations of sleep duration with mortality risk were examined using Cox’s proportional hazard models with the procedure in SPSS^®^ software version 11.0.^12^ We computed hazard ratios and their 95% confidence intervals adjusted for age at baseline alone, as well as those adjusted for age, SBP, total cholesterol, BMI, smoking habits, alcohol drinking habits, education, and marital status.

## RESULTS

The total observed person-years were 93,424, and the mean follow-up period±standard deviation(SD) was 8.2±1.5 years. The mean ages±SD were 55.0±11.9 years for males and 55.2±11.2 years for females at baseline data collection. There were 495 deaths (4.3%) during the study period: 289 males (6.5%) and 206 females (3.0%).

At baseline, 3% of the males and 4% of the females reported to sleep for shorter than 6 hours/day, 11% and 16% for 6-6.9 hours/day, 29% and 37% for 7-7.9 hours/day, 37% and 32% for 8-8.9 hours/day, and 20% and 12% for 9 hours/day or longer. Means±SD of sleep duration were 7.8±1.7 hours in males and 7.4±1.1 hours in females (p<0.001). Baseline characteristics in relation to sleep duration are shown in Table 1. The mean age, SBP, and DBP tended to increase with sleep duration in both sexes. Longer sleepers of both sexes were more likely to less educated than those who slept shorter than 8 hours.

**Table 1.** Baseline characteristics according to sleep duration.

Sex		Sleep duration (hours/day)									

		-5.9		6.0-6.9		7.0-7.9		8.0-8.9		9.0-	
Males	n (%)	125	(3)	481	(11)	1264	(29)	1651	(37)	898	(20)
	Number of death (% of total number)	16	(12.8)	24	(5.0)	62	(4.9)	94	(5.7)	93	(10.4)
	Age (year)	53.1	(14.7)	51.3	(11.8)	52.5	(11.8)	55.2	(11.5)	60.3	(10.7)
	Systolic blood pressure (mmHg)	130.4	(18.8)	129.5	(20.7)	129.6	(19.9)	131.3	(20.4)	135.2	(21.3)
	Diastolic blood pressure (mmHg)	78.2	(11.8)	78.1	(12.9)	78.3	(12.3)	79.2	(12.2)	81.0	(12.0)
	Total cholesterol (mg/dL)	184.6	(33.7)	184.4	(34.3)	186.3	(34.4)	186.1	(33.7)	181.6	(34.9)
	High density lipoprotein cholesterol (mg/dL)	48.5	(12.6)	47.5	(12.5)	48.9	(12.1)	48.9	(13.3)	49.7	(13.2)
	Body mass index (kg/m^2^)	22.9	(2.8)	23.0	(2.9)	23.1	(2.8)	23.0	(2.9)	22.7	(2.9)
	Current smokers (%)	55.6		54.8		50.5		52.6		45.9	
	Current alcohol drinkers (%)	72.6		72.7		75.1		78.4		72.2	
	Age (year) at finished education (%)										
	<15	12.4		8.2		8.2		11.2		22.7	
	15-17	47.9		49.0		51.0		50.8		55.8	
	18+	39.7		42.7		40.8		37.9		21.5	
	Having a spouse (%)	89.5		91.6		85.8		92.9		91.8	

Females	n (%)	254	(4)	1112	(16)	2529	(37)	2211	(32)	800	(12)
	Number of death (% of total number)	4	(1.6)	28	(2.5)	55	(2.2)	68	(3.1)	51	(6.4)
	Age (year)	52.4	(10.4)	52.3	(10.8)	53.3	(10.9)	57.1	(10.9)	61.4	(10.2)
	Systolic blood pressure (mmHg)	123.2	(17.8)	125.4	(20.6)	126.9	(21.1)	129.4	(20.8)	133.8	(21.7)
	Diastolic blood pressure (mmHg)	74.1	(10.3)	75.2	(12.2)	75.7	(12.3)	76.8	(11.9)	78.7	(11.9)
	Total cholesterol (mg/dL)	193.5	(36.3)	194.3	(35.7)	196.0	(35.4)	198.5	(33.8)	200.5	(34.0)
	High density lipoprotein cholesterol (mg/dL)	53.2	(12.9)	53.4	(12.9)	52.9	(12.4)	52.5	(12.3)	51.2	(12.6)
	Body mass index (kg/m^2^)	23.2	(3.2)	23.4	(3.1)	23.0	(3.1)	23.1	(3.2)	23.5	(3.5)
	Current smokers (%)	8.8		6.7		5.5		4.9		4.6	
	Current alcohol drinkers (%)	26.8		28.9		25.9		23.4		20.7	
	Age (year) at finished education (%)										
	<15	13.5		12.0		14.4		25.3		41.1	
	15-17	48.8		47.9		48.0		49.4		43.4	
	18+	37.7		40.1		37.6		25.3		15.6	
	Having a spouse (%)	93.7		94.1		93.3		90.7		86.6	

Mean values (standard deviation) for quantitative data and proportion in percentage for qualitative data are shown.

Table 2 presents crude mortality rate and the two kinds of hazard ratios for all-cause mortality by sleep duration. Crude mortality increased in males who slept shorter than 6 hours/day and 9 hours or longer, and in females who slept 9 hours or longer. After adjustment for age, a reverse J-shaped relationship was observed between mortality and sleep duration among males. Especially males sleeping shorter than 6 hours/day had significantly higher risk than those sleeping 7-7.9 hours. Females sleeping 9 hours or longer also had higher risk, but females sleeping shorter than 6 hours did not show such an increased risk. After further adjustment for SBP, total cholesterol, BMI, smoking habits, alcohol drinking habit, education, and marital status, the results remained essentially unaltered.

**Table 2.** Association between sleep duration and all-cause mortality: crude mortality rate and adjusted hazard ratios.

Sex	Sleep duration (hours/day)			Crude mortality rate (per 1000 person-years)	HR-age (95% confidence interval)	HR-all (95% confidence interval)
Males		-	5.9	15.6	2.3 (1.3-3.9)	2.4 (1.3-4.2)
	6.0	-	6.9	6.0	1.1 (0.7-1.8)	1.1 (0.7-1.8)
	7.0	-	7.9	5.9	1.0 (reference)	1.0 (reference)
	8.0	-	8.9	6.8	1.0 (0.7-1.3)	0.9 (0.6-1.2)
	9.0	-		12.7	1.3 (0.9-1.8)	1.1 (0.8-1.6)

Females		-	5.9	1.9	0.8 (0.3-2.2)	0.7 (0.2-2.3)
	6.0	-	6.9	3.1	1.3 (0.8-2.0)	1.3 (0.8-2.1)
	7.0	-	7.9	2.6	1.0 (reference)	1.0 (reference)
	8.0	-	8.9	3.7	1.0 (0.7-1.5)	1.1 (0.8-1.6)
	9.0	-		7.9	1.5 (1.0-2.3)	1.5 (1.0-2.4)

HR-age : hazard ratios adjusted for age.

HR-all : hazard ratios adjusted for age, systolic blood pressure, total cholesterol, body mass index, smoking habits, alcohol drinking habits, education, and marital status.

Table 3 demonstrates the cause-specific hazard ratios. Males sleeping shorter than 6 hours and females sleeping 9 hours or longer were at significantly higher risk of death from heart diseases than those sleeping 7-7.9 hours. The risk of death from malignant neoplasms and external causes was significantly higher for males sleeping shorter than 6 hours.

**Table 3.** Association between sleep duration and cause specific mortality (ICD-10): adjusted hazard ratios.

Sex	Sleep duration (hours/day)			heart diseases (I10-I15,I20-I25,I27,I30-I52)		stroke (I60-I69)		malingant neoplasms (C00-C97)		external causes (V01-Y89)		others	
				No. of death	Hazard ratio* (95% CI)	No. of death	Hazard ratio* (95% CI)	No. of death	Hazard ratio* (95% CI)	No. of death	Hazard ratio* (95% CI)	No. of death	Hazard ratio* (95% CI)
Males		-	5.9	3	6.2 (1.3-29.5)	1	1.3 (0.2-11.0)	7	3.1 (1.3-7.5)	4	3.1 (1.0-9.7)	1	†
	6.0	-	6.9	6	3.8 (1.0-14.7)	2	0.8 (0.2-3.9)	4	0.6 (0.2-1.6)	5	0.9 (0.3-2.7)	7	1.4 (0.6-3.7)
	7.0	-	7.9	5	1.0 (reference)	8	1.0 (reference)	21	1.0 (reference)	15	1.0 (reference)	13	1.0 (reference)
	8.0	-	8.9	3	0.4 (0.1-1.9)	4	0.2 (0.1-0.8)	51	1.4 (0.8-2.4)	11	0.6 (0.3-1.4)	25	1.0 (0.5-2.0)
	9.0	-		9	1.3 (0.4-4.5)	19	1.2 (0.5-3.0)	32	1.3 (0.7-2.3)	6	0.5 (0.2-1.4)	27	1.3 (0.7-2.6)

Females		-	5.9	0	-	0	-	3	1.1 (0.3-4.7)	0	-	1	1.1 (0.1-8.5)
	6.0	-	6.9	0	-	6	3.2 (1.0-10.5)	15	1.5 (0.7-3.0)	5	1.0 (0.3-3.1)	2	0.3 (0.0-2.4)
	7.0	-	7.9	3	1.0 (reference)	6	1.0 (reference)	24	1.0 (reference)	11	1.0 (reference)	11	1.0 (reference)
	8.0	-	8.9	10	7.3 (0.9-58.6)	9	1.4 (0.4-4.3)	30	1.2 (0.7-2.1)	6	0.5 (0.2-1.4)	13	0.9 (0.4-2.1)
	9.0	-		15	15.9 (2.0-128.8)	8	2.5 (0.8-8.2)	14	1.1 (0.5-2.3)	3	0.4 (0.1-2.0)	11	1.3 (0.5-3.4)

* : Hazard ratios adjusted for age, systolic blood pressure, total cholesterol, body mass index, smoking habits, alcohol drinking habits, education, and marital status.

CI : confidence interval

† : uncalculable because of only one death in this category whoes data about education are missing.

- : no deaths in the category.

To eliminate the influence of potentially preexisting sub clinical diseases, we repeated the analyses excluding those who died within 2 years after the baseline examination. The results were almost identical (data not shown).

## DISCUSSION

We investigated the association between reported sleep duration and mortality using data from the Jichi Medical School Cohort Study, a population-based cardiovascular cohort study. The present study showed a reverse J-shaped relationship between reported sleep duration and mortality in males, with a remark of males sleeping shorter than 6 hours/day showed a significantly higher risk. In females, however, only when sleeping 9 hours or longer had a higher risk.

Many previous studies,^1^^-^^6^ though not all,^7^^-^^10^ reported that both long and short sleep duration increased risk of death. However, there have been only a few studies controlling for various relevant confounding factors.^4^^-^^7^ According to Kripke et al. studying in a large cohort of the Cancer Prevention Study II,^4^ both males and females, who slept around 7 hours yielded best survival. Participants who reported to sleep for 8 hours or longer had a significantly increased risk of death, as did those who slept 6 hours or shorter. In a recently published prospective study in West Jerusalem, males who slept longer than 8 hours had a substantially elevated risk of all-cause mortality, but there was no significant association of sleep duration with mortality in females.^7^

In Japan, Kojima et al. studied the effect of sleep durations using a population-based cohort in Gifu Prefecture.^5^ Both long and short sleep, compared to 7-8 hour-sleep, was related to significantly increased risk of all-cause mortality in males, but not in females. Recently, Tamakoshi et al. found in the Japan Collaborative Cohort Study for Evaluation of Cancer Risk that both long and short sleep durations increased all-cause mortality and those who slept 7 hours were associated with the best survival.^6^ The present study further confirmed these relationships among males.

We cannot explain why there was difference between the result of males and that of females in our study. There was no other study of which result was same as ours. The characteristic of our study was the subjects inhabited in rural areas in Japan. It may influence our result, but we do not know what influence.

Several explanations for associations between decreased sleep duration and higher mortality have been postulated. Short-term sleep deprivation caused impaired glucose tolerance, higher evening cortical levels, activation of the sympathetic nervous system, increased blood pressure, reduced leptin levels, and increased inflammatory markers.^13^^-^^16^ Although the magnitude of the physiologic changes found in these short-term studies was modest, they provide a potential mechanism whereby long-term sleep restriction may affect long-term health. In contrast, there was no evidence suggesting that increased sleep duration would deteriorate life.

We examined whether sleep duration is associated with cause-specific mortality. In our study, males sleeping shorter than 6 hours and females sleeping 9 hours or longer were at significantly higher risk of death from heart diseases than those sleeping 7-7.9 hours. Because the number of deaths in our study was not enough, the power to detect differences between 7-7.9 hour goops and other groups was limited. There were only two studies examining the relationship between sleep duration and cause-specific mortality.^2^^,^^3^ Both studies did not control for various relevant confounding factors. Further investigations would be required to resolve the relation between sleep duration and cause-specific mortality.

Strength of our study is that we controlled for biomedical factors such as blood pressure, serum lipid, and obesity that may influence mortality. Other studies adjusted for solely BMI,^4^^,^^6^ or BMI, SBP and total cholesterol.^7^ We showed the hazard ratios adjusted for SBP, total cholesterol, and BMI simultaneously, although the results were similar to that from univariate analyses.

There are several limitations in the present study. First, sleep duration and other potential risk factors were based on self-report by the participants. Questions about self-reported sleep duration were demonstrated in other studies,^17^^,^^18^ to be valid measures in comparison with quantitative sleep assessments by actigraphy. In the current study, we asked about whole-day activities and calculated sleep duration. Therefore the data were deemed to be more accurate than those obtained by simply asking about sleep duration. Second, we had no information about the quality of sleep in our cohort study. Several studies reported that the quality of sleep was related to mortality.^3^^,^^5^^,^^9^^,^^19^ Indeed, there is neither clear definition nor established measure of the quality of sleep, and some studies indicated that insomnia was not associated with increased mortality.^4^^,^^10^^,^^20^^,^^21^ The third limitation is that background psychological factors were not considered. Sleep patterns are known to be associated with psychological factor.^22^^-^^24^ In the Japan Collaborative Cohort Study for Evaluation of Cancer Risk, the U-shaped association between sleep duration and mortality among males changed to a J-shaped association after adjusting for mental conditions (they chose perceived mental stress and depressive symptoms).^6^ Further studies are needed. Finally, the reasons of short sleep (insomnia, over work, or watching television) were not ascertained in our study. These factors might affect the risk of death.

In conclusion, our data indicated that men sleeping less than 6 hours/day and women sleeping 9 hours or more had significantly higher risk of death than those sleeping 7-7.9 hours. Optimal sleep duration for human health was suggested.
